# The expression of fungal CotH, human glucose-regulated protein 78 (GRP78), and predicted miRNAs in macrophages and diabetic mice infected with *Rhizopus oryzae*

**DOI:** 10.1128/spectrum.02852-24

**Published:** 2025-06-09

**Authors:** Zahra Seifi, Tahereh Shokohi, Mohammad Shafiee, Seyed Javad Mowla, Farhad Niknejad, Emmanuel Edwar Siddig, Ayman Ahmed, Mahdi Abastabar, Laleh Vahedi Larijani

**Affiliations:** 1 Student Research Committee, Mazandaran University of Medical Sciences 92948, Sari, Iran; 2 Laboratory Sciences Research Center, Golestan University of Medical Sciences 125691, Gorgan, Iran; 3 Invasive Fungi Research Center, Communicable Diseases Institute, Mazandaran University of Medical Sciences 92948, Sari, Mazandaran Province, Iran; 4 Department of Medical Mycology, School of Medicine, Mazandaran University of Medical Sciences 92948, Sari, Iran; 5 Stem Cell Research Center, Golestan University of Medical Sciences 125691, Gorgan, Iran; 6 Department of Medical Genetics, School of Advanced Technologies in Medicine, Golestan University of Medical Sciences 125691, Gorgan, Iran; 7 Department of Molecular Genetics, Faculty of Biological Sciences, Tarbiat Modares University 41616, Tehran, Iran; 8 Department of Medical Parasitology and Mycology, Faculty of Paramedicine, Golestan University of Medical Sciences 125691, Gorgan, Iran; 9 Unit of Basic Medical Sciences, Faculty of Medical Laboratory Sciences, University of Khartoum 89235, Khartoum, Sudan; 10 Mycetoma Research Center, University of Khartoum 89235, Khartoum, Sudan; 11 Department of Medical Microbiology and Infectious Diseases, ErasmusMC, University Medical Center Rotterdam 6993, Rotterdam, South Holland, the Netherlands; 12 Institute of Endemic Diseases, University of Khartoum 89235, Khartoum, Sudan; 13 Swiss Tropical and Public Health Institute (Swiss TPH) 30247 , Allschwil, Switzerland; 14 University of Basel 27209, Basel, Basel-Stadt, Switzerland; 15 Department of Pathology, School of Medicine, Mazandaran University of Medical Sciences 92948, Sari, Iran; Ruhr-Universitat Bochum, Bochum, Germany

**Keywords:** mucormycosis, *Rhizopus oryzae*, macrophages, diabetic mice, CotH protein, miRNAs

## Abstract

The spore coat protein homolog (CotH) has been identified among virulence factors of Mucorales that mediate fungal invasion by glucose-regulated protein 78 (GRP78) ligand on endothelial cells. The aim of this study was to examine the gene expressions of spore coat protein homolog 3 (CotH3) and GRP78, along with their target miRNAs, in infected human macrophages, mice models, and mucormycosis patients. To assess changes in the relative expressions of the GPR78 and CotH3 genes, this study used the real-time quantitative PCR method to quantify their target miRNAs in macrophages derived from human monocytes (monocyte-derived macrophages [MDMs]), mice models, and sinus tissue from diabetic patients with mucormycosis. In this study, expressions of GRP78 and CotH3 genes were elevated in infected MDMs and non-diabetic Balb/c mice infected with *Rhizopus oryzae*, and the expression of the GRP78 gene was upregulated in the non-infected diabetic mice. However, the infected diabetic mice displayed a decline in the GRP78 gene expression. Moreover, although the expression of the GRP78 gene increased in the sinus tissue of diabetic patients with mucormycosis, it significantly decreased in the same patients after treatment. The relative expression of hsa-miR-16–5p, hsa-miR-93–3p, and hsa-miR-335–5p was downregulated in the MDMs. The increased gene expression levels of CotH3 and GPR78 observed in infected macrophages provide insight into the mechanism of interactions between the pathogen and macrophages. Prolonged encounters lead to changes in the expression levels of CotH3 and GPR78 genes.

**IMPORTANCE:**

The research delves into the intricate gene expression patterns of CotH3, a homolog of CotH, and GRP78 in human macrophages, mice models, and diabetic patients afflicted with mucormycosis. The study's findings underscore the pivotal role of diabetes in the host-pathogen interaction, revealing that diabetic conditions amplify the expression of the GRP78 gene, thereby escalating the risk of fungal invasion and growth. This research paper is crucial as it sheds light on the intricate mechanisms underlying mucormycosis infection and underscores the heightened vulnerability of diabetic individuals. By elucidating the roles of CotH3 and GRP78 in the infection process, the study contributes to a deeper understanding of mucormycosis pathogenesis and paves the way for the development of targeted therapeutic strategies.

## INTRODUCTION

Mucormycosis is a life-threatening infection that has become more prevalent among immunocompromised patients, diabetic patients with ketoacidosis, and immunocompetent individuals who have been exposed to contaminated soil following trauma. The disease is characterized by rapid local spread, tissue necrosis, and angioinvasion and is caused by filamentous fungi of the Mucorales order. Approximately 70%–80% of all causative pathogens among the Mucorales belong to the genera *Rhizopus*, *Lichtheimia* (formerly *Absidia*), and *Mucor* (1–3). Mucormycosis, with *Rhizopus oryzae* being the most common cause, is responsible for about 70% of these infections. Interestingly, in recent years, due to the rising prevalence of diabetes, malignancy, organ transplantation, and climate change, the number of individuals at risk of acquiring this infection is significantly rising (4). Despite surgical debridement and adjunctive antifungal therapy, the overall mortality rate of mucormycosis remains dangerously high at approximately 50% and can approach 100% in patients with disseminated disease or persistent neutropenia, mainly attributed to the delay in detecting the infection (2, 4, 5). Therefore, there is a critical need to improve diagnostic capacities and to develop new strategies to prevent and treat this disease, particularly in endemic and high-risk countries.

Infection takes place through various routes such as inhalation, ingestion of contaminated food, or through skin breaks or abrasions allowing the pathogen introduction into the body from soil, air, or water contaminated with infectious spores (5). Depending on the route of introduction, the pathogen causes several different clinical manifestations, including rhino-orbital/cerebral, pulmonary, gastrointestinal, or cutaneous infections (6). One common feature of mucormycosis, regardless of the type of infection, is the highly aggressive and rapid invasion of blood vessels by the causative organism (6). This leads to hematogenous dissemination, vessel thrombosis, and tissue necrosis. As a result, the interactions between invading fungi and endothelial cells are one of the main factors in the pathogenesis of mucormycosis.

Although the predisposing factors may vary, the invasion of vasculature leading to blood vessel thrombosis and subsequent tissue necrosis is a characteristic of mucormycosis caused by all Mucorales (7). Therefore, the interaction between fungi and the endothelial cells lining the vasculature is a crucial step in the pathogenesis of this infection. Recently, studies have shown that *R. oryzae* strains are capable of adhering to human umbilical vein endothelial cells and inducing endocytosis (8).

Research has also identified glucose-regulated protein 78 (GRP78) as the endothelial cell receptor used by Mucorales during the invasion of the host’s cell. High concentrations of glucose and iron, as seen during hyperglycemia, diabetic ketoacidosis, or acidosis, cause an increase in GRP78 expression, enabling fungal invasion and damage of endothelial cells in a receptor-dependent manner (9). Interestingly, numerous studies demonstrated that the spores of *Rhizopus* spp. germinate in hyperglycemic and hyperketonemic states and induce the invasion of human umbilical vein endothelial cells (HUVECs) through interactions between the fungal ligands known as spore coat protein homologs (CotHs) and GPR78 (8–10). Furthermore, studies have shown that spore coat protein homolog 3 (CotH3) plays a crucial role in fungal-host invasion, and the CotH protein family is unique to Mucorales and absent in other pathogens (10, 11). In general, elevated levels of glucose, iron, and ketone bodies that are observed in patients with hyperglycemia and diabetic ketoacidosis may increase the expression of GRP78 and CotH3, resulting in facilitating the fungal penetration and damage to HUVECs (10, 12).

Growing evidence demonstrates that the inhibition of fungal invasion of host cells, both *in vitro* and *in vivo*, can be achieved by manipulating the expression of CotH3 through genetic or immunological-inhibition methods (7, 13).

MiRNAs, also known as microRNAs, are small RNA molecules measuring around 19–24 nucleotides in length. Despite being non-coding, they are widely present in the genomes of both animals and humans, and they are characterized by being conserved throughout evolution. Previous research has also suggested that miRNAs can regulate gene expression at a post-transcriptional level through a process that is referred to as post-transcriptional modifications (13). It plays a crucial role in numerous cellular processes such as apoptosis, cell differentiation, and proliferation (14). Recent studies have revealed that miRNAs play a significant role in fungal infections (15–18), including the regulation of antifungal immune response, since the miRNAs that are present in host cells can regulate the immune response against fungal infections (13). For instance, miR-155 has been shown to play a role in regulating the expression of inflammatory cytokines against *Candida albicans* infection (14). Furthermore, miRNAs also play a role in the modulation of fungal virulence. Studies demonstrated that miRNA-like molecules secreted by *Aspergillus fumigatus* can alter the gene expression profiles of host epithelial cells and induce inflammatory responses (15).

Furthermore, miRNAs are involved in modulating macrophage function and the innate immune response (16). The miRNAs hsa-miR-16–5p, hsa-miR-93–3p, hsa-miR-335–5p, and mmu-miR-181b-5p have been implicated in both fungal infections and macrophage function (17). Hsa-miR-16–5p and hsa-miR-93–3p have been found to regulate the immune response to fungal infections and macrophage polarization, with hsa-miR-16–5p inhibiting M2 polarization and promoting a pro-inflammatory M1 phenotype, while hsa-miR-93–3p promotes an anti-inflammatory M2 phenotype (18). Meanwhile, hsa-miR-335–5p targets genes involved in regulating macrophage phenotypes and toll-like receptor signaling in response to fungal infections. Moreover, mmu-miR-181b-5p has been shown to regulate macrophage phagocytosis and intracellular trafficking, with potential therapeutic implications for both fungal infections and inflammatory diseases (19).

Additionally, computational and bioinformatics analysis have suggested that hsa-miR-16–5p affects the ATK gene as a serine/threonine-protein kinase involved in the regulation of cell survival, glucose metabolism, cell proliferation, apoptosis, insulin signaling, angiogenesis, and tumor growth. Interestingly, ATK is associated with the GPR78 expression signaling pathway, resulting in apoptosis suppression (20). It is well established that macrophages recognize pathogens and internalize them through phagocytosis, and increased expression of hsa-miR-16–5p, hsa-miR-93–3, and hsa-miR-335–5p has an essential role in regulating macrophage functions. In addition, this cluster of miRNAs controls intracellular signaling pathways (e.g., TLR and MyD88 signaling pathways) by influencing the expression of transcription factors (21).

Considering that, understanding the various aspects of the interplay between hosts and pathogens during infection is important for developing effective preventive, control, and case management measures including drugs and vaccines (22). Therefore, the objective of this study was to assess the patterns of gene expression of CotH3, GPR78, and their target miRNAs (hsa-miR-16–5p, hsa-miR-93–3p, hsa-miR-335–5p, and mmu-miR-181b-5p) during infection in macrophages derived from human monocytes (monocyte-derived macrophages [MDMs]), renal tissue of diabetic BALB/c mice infected with *R. oryzae*, with and without treatment with liposomal amphotericin B (LAmB), as well as in sinus tissue from patients with mucormycosis.

## MATERIALS AND METHODS

### MDMs

In order to harvest and refine human macrophages, monocytes were retrieved from peripheral blood samples of healthy individuals using Ficoll-Paque (biowest, France) according to the manufacturer. The retrieved monocytes were then placed in Roswell Park Memorial Institute (RPMI) 1640 medium with Glutamax and supplemented with 10% heat-inactivated fetal calf serum, along with 100 U/mL of penicillin and streptomycin, separately. The cells were then incubated at 37°C with 95% air and 5% CO_2_ for a period of 2 weeks to complete the incubation process (23). Macrophages were exposed to hyphae for different incubation times. Untreated hyphae served as control. The macrophages-fungus contact was evaluated using an inverted microscope (Olympus, USA).

### Co-culturing MDMs with fungal hyphae

The CBS 112.07 strain of *R. oryzae* was grown in malt extract agar (MEA) at a temperature of 28°C for a period of 3–5 days. Following the spore germination in RPMI 1640, MDMs were subjected to 1 × 10^7^ of *R. oryzae* or Mucorales, which were added to each dish in such a way that there were approximately 1.5 cells for every one human cell for a multiplicity of infection. This mix of cells was then incubated for 6–16 h (11). An inverted microscope from Olympus was utilized to differentiate between the non-infected and infected MDMs. The non-infected MDMs were used as a control.

### Preparation of animal experiments and clinical samples

**Fig 1:**
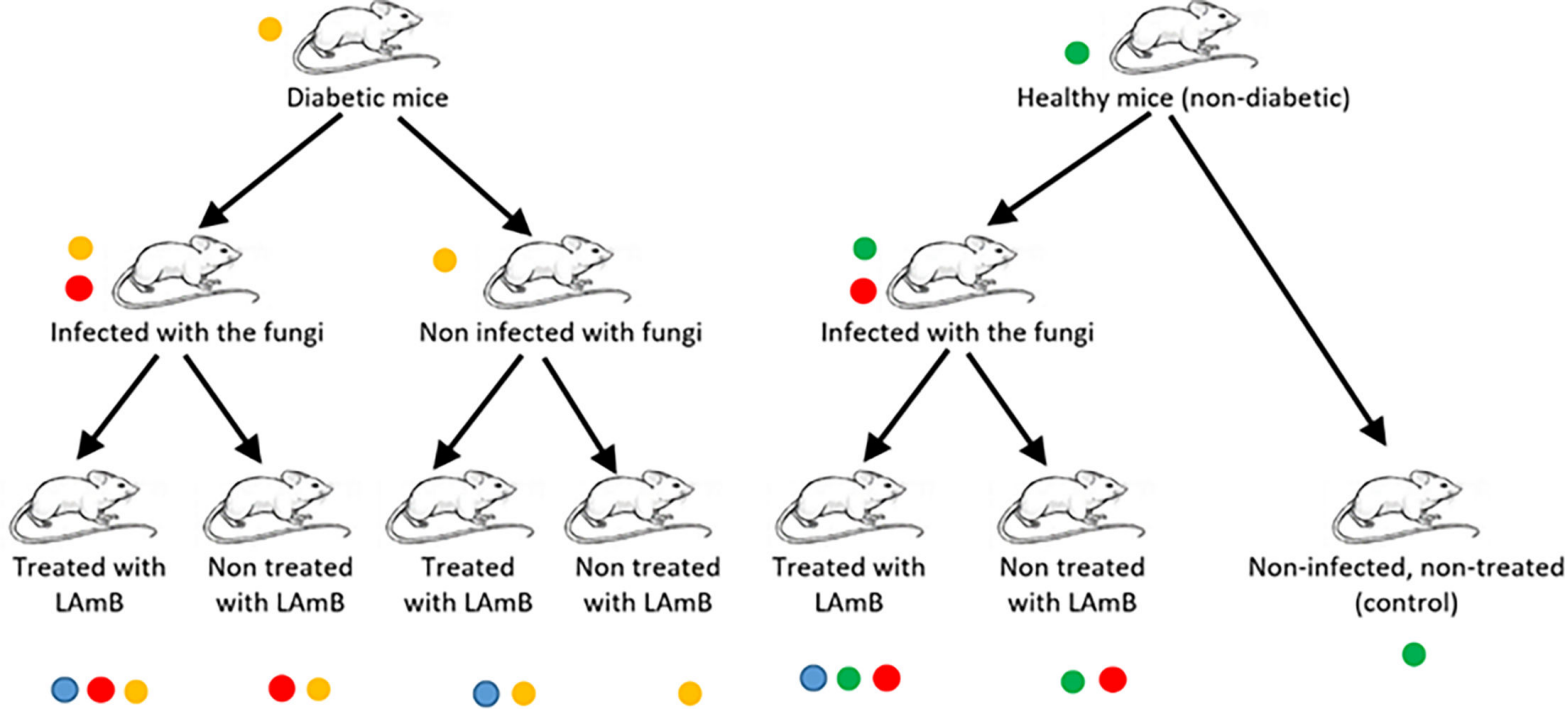
Flowchart explaining the mice grouping.

The *in vivo* study was conducted on male BALB/c mice, aged between 4 and 6 weeks and weighing between 21.0 and 21.7 g. These mice were acquired from the Institute for Laboratory Animal Research, Mazandaran University of Medical Sciences, Sari, Iran, and were provisioned with water and food *ad libitum*. They were randomly allocated into seven groups, with five mice per cage. The groups were categorized as non-diabetic, non-infected, and non-treated (control); non-diabetic, infected, and non-treated; non-diabetic, infected, and treated with LAmB; diabetic, non-infected, and non-treated; diabetic, non-infected, and treated with LAmB; diabetic, infected, and non-treated; and diabetic, infected, and treated with LAmB (Fig. 1).

### Diabetic ketoacidotic mouse model

For diabetic mice preparation, diabetes was induced in mice after 4 h of fasting by a single intraperitoneal injection of streptozotocin (STZ) 20 mg/mL (210 mg/kg [1 mg/100 g]; Santa Cruz, USA) in 0.2 mL of sodium citrate buffer (pH 4.5) within 10 days before the infection (24). Diabetes was confirmed by a tail-blood glucometer 72 h after STZ administration. Mice with blood glucose levels of 250 mg/dL or greater were considered diabetic (25). Similarly, the experiment was conducted with a blood glucose level of 90–120 mg/dL in the control group.

### Primary disseminated mucormycosis

To infect mice, a volume of 200 µL with 10^5^ µL per mouse was counted as previously described and prepared in physiological serum for inoculation by intravenous lateral venous injection into non-control mice (26). After infection, the mice were randomly sorted into different treatment groups. The treated group was under treatment with LAmB (15 mg/kg/day) (from a stock of 4 mg/mL) for 4 days (27). To determine the efficacy and the minimum inhibitory concentrations (MIC) of LAmB on *R. oryzae* (CBS 112.07) studied, the MIC of LAmB medication in the group of treated mice was estimated after 48 h using the protocol of the Clinical and Laboratory Standards Institute (M38-3rd ed) (28). Histopathological assessments were carried out on sections of the harvested kidneys after fixing in 10% formalin. The fixed organs were embedded in paraffin, and 5 µm sections were stained with periodic acid-Schiff to detect *R. oryzae* hyphae.

In this study, clinical samples from cases of rhinocerebral mucormycosis were obtained from two diabetic patients to serve as the human mucormycosis model. Clinical samples were collected from the necrotic tissues of the infected patient, and additional samples were collected from the intact border of defective tissues from the same persons to serve as a control. Pre- and post-treatment specimens were obtained from the patient before and after treatment with LAmB. The level of fasting blood sugar for treated and non-treated patients was 250 and 400 mg/dL, respectively. The tissue culture was done in MEA media following a high-aseptic technique and incubated at 28°C for 1 week.

### RNA isolation

Total RNAs were extracted from all the cells under experiment including MDMs, clinical samples, and mice kidney tissue, by One Step-RNA solution from Bio Basic Inc (Markham, Canada) according to the manufacturer’s instruction. The quality and quantity of extracted RNA were evaluated using 2% agarose gel and a nanodrop spectrophotometer instrument (Biochrome Ltd, Cambridge, UK) (The goal is to observe sharp 18S and 28S rRNA bands. The apparent ratio of 28S to 18S should also be 2:1). RNA was treated with DNase I (CinnaGen Co, Iran) to remove any potential DNA residuals. For quality control, the RNA purity was measured at A260/A280 nm absorbance ratio, and the concentration was quantified using a spectrophotometer (A260/A280 = ~2.0 is accepted). Purified total RNA was stored at −80°C until later use.

### cDNA synthesis

First-strand cDNA was synthesized using a Revert Aid First Strand cDNA Synthesis Kit (Thermo Scientific, Lithuania) according to the manufacturer’s instruction. To amplify the cDNA, specific primers provided by Genfanavaran Corporation for each miRNA and 5S rRNA primers as control genes were used according to the miR-Q method (29). Further information about the oligonucleotide sequences used for cDNA synthesis and amplification of miRNAs is presented in Table 1. For each sample, a no- reverse transcriptase control was used in parallel with the DNase-treated RNA to detect any potential non-specific genomic DNA amplification.

**TABLE 1:** Oligonucleotide sequences were used for cDNA synthesis and amplification of miRNAs

Oligonucleotide name	Sequences
RT6^*a*^-335 Short^*b*^-335	TGTCAGGCAACCGTATTCACCGTGAGTGGTACATTT CGTCAGATGTCCGAGTAGAGGGGGAACGGCGTTCAAGAGCAATAACGAA
RT6-93 Short-93	TGTCAGGCAACCGTATTCACCGTGAGTGGTCGGGAA CGTCAGATGTCCGAGTAGAGGGGGAACGGCGTACTGCTGAGCTAGCAC
RT6-16 Short-16	TGTCAGGCAACCGTATTCACCGTGAGTGGTCGCCAA CGTCAGATGTCCGAGTAGAGGGGGAACGGCGTTAGCAGCACGTAAAAA
RT6-181b Short-181b	TGTCAGGCAACCGTATTCACCGTGAGTGGTAACCCA CGTCAGATGTCCGAGTAGAGGGGGAACGGCGTAACATCATTGCTGTCGG
MP-F^*c*^ MP-R^*d*^	TGTCAGGCAACCGTATTCACC CGTCAGATGTCCGAGTAGAGG

^*a*^ RT6 oligonucleotide primer-specific miRNA.

^*b*^ PCR oligonucleotide primer-specific miRNA.

^*c*^ Forward universal primer.

^*d*^ Reverse universal primer.

### Quantitative reverse transcriptase polymerase chain reaction

Quantitative reverse transcriptase polymerase chain reaction (Q-RT-PCR) was performed with the Step One Plus Real-Time PCR System (Applied Biosystems, Foster City, USA) using qRT-PCR SYBR Green Master Mix (Fermentas Company, Thermo Scientific, Waltham, MA, USA) according to the manufacturer’s instruction (Thermo Scientific). First, by performing bioinformatics analyses in the miRWalk two database, it identifies validated and predicted microRNAs that target GRP78 completely in different conditions and tissues, according to the required selection. In the present study, expression levels of CotH3, Actin, GRP78, 5S rRNA, and GAPDH genes, as well as hsa-miR-335–5p, hsa-miR-16–5p, hsa-miR-93–3p, and mmu-miR-181b-5p miRNAs, were evaluated in macrophages derived from human monocytes, clinical samples, and kidney tissue of mice. The nucleotide sequences of miRNAs were obtained from miRWalk 2 and www.mirbase.org as primer sequences for amplifying the cDNA of CotH3, GRP78 (mice and human), 5S rRNA, GAPDH, and β-actin (as an internal control) genes are listed in Table 2. The product lengths were 126 (151 and 222), 114, 249, and 121 bp, respectively, with the annealing temperature of 58°C. To compensate for variations in the amount of input RNA and the efficacy of reverse transcriptase, β-actin mRNA was quantified as an internal control, and all gene expressions were accordingly normalized. Non-template controls for each primer set were assayed for potential DNA contamination or primer dimerization. The specificity of each pair of primers was confirmed by BLAST software, and to assess the expression of the target miRNA genes and design their primers, the miR-Q method (29) was used (Tables 1 and 2). The primers were applied at 10 pmol/µL concentrations. All experiments were performed at least in duplicate using a thermal cycler under the following conditions: 95°C for 15 seconds, 65°C for 15 seconds, and 60°C for 1 min. Melting curves were then determined with temperatures ranging from 65°C to 95°C. To calculate the relative fold change of each gene, we used the 2^−ΔΔCt^ method.

**TABLE 2:** Gene symbol, sequence of forward (F) and reverse (R) primers, and amplicon length of genes

Gene	Primer	Sequence (5′→3′)	Fragment length (bp)
GRP78^*a*^ (human)	F	GGAAAGAAGGTTACCCATGC	222
	R	AGAAGAGACACATCGAAGGT	
GRP78^*b*^ (mice)	F	TCTTGCCATTCAAGGTGGTTG	151
	R	TTCTTTCCCAAATACGCCTCAG	
CotH3^*c*^	F	GCCAATCCTAATGGTGAAGC	126
	R	CATGAAACGGTCGAGATCAA	
GAPDH^*d*^	F	ACCATCTTCCAGGAGCGAG	249
	R	TAAGCAGTTGGTGGTGCA	
ACT^*e*^	F	AGCTCCTTTGAACCCCAAGT	121
	R	ACGACCAGAGGCATACAAGG	
5S rRNA^*f*^	F	GCCCGATCTCGTCTGATCT	114
	R	AGCCTACAGCACCCGGTATT	

^*a*^ GRP78 (human), glucose-regulated protein 78.

^*b*^ GRP78 (mice), glucose-regulated protein 78 (*Mus musculus*).

^*c*^ CotH3, spore coat protein homolog 3.

^*d*^ GAPDH, glyceraldehyde-3-phosphate dehydrogenase.

^*e*^ ACT, β-actin.

^*f*^ 5S ribosomal RNA.

### Statistical analysis

Data are presented as mean values, and error bars are indicated as ±SEM. The significance of differential expression was analyzed using the *t*-test and analysis of variance. Q-RT-PCR data were adjusted based on the exact PCR efficiency. SPSS software package version 22 was used for all statistical comparisons and analyses. Statistically, *P* < 0.05 was considered significant.

## RESULTS

### RNA isolation

The optical density 260/280 of extracted total RNA of all samples was in the range of 1.8–2.0, and the concentration was 100–1,600 ng. In qualitative analysis on 2% agarose gel, 28S, 18S, and 5S ribosomal RNA bands were observed in the samples (RNA quality photo, Fig. S1; DOI: 10.6084/m9.figshare.28513859, https://figshare.com/account/items/28513859/edit).

#### MDM aggregation around the hyphae

**Fig 2:**
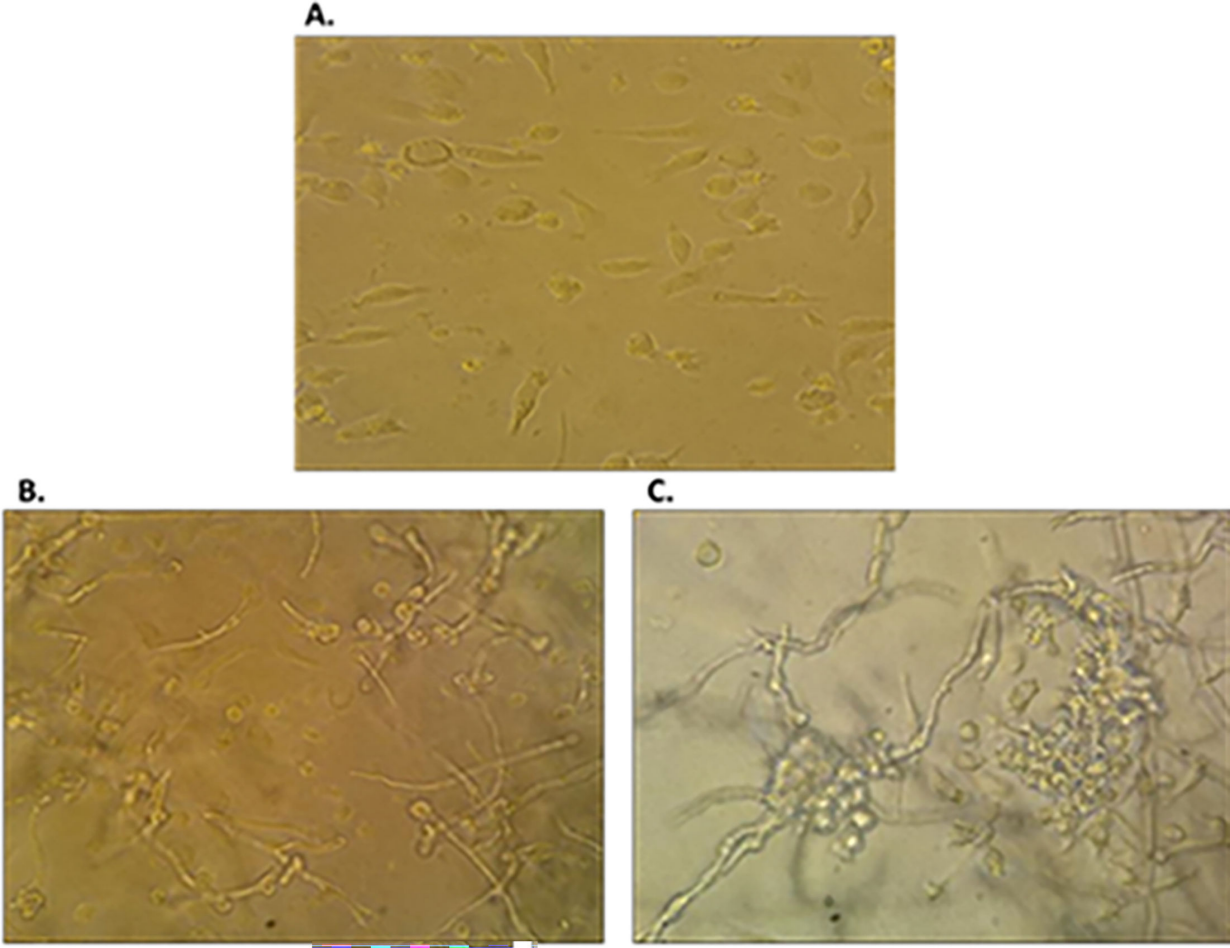
Control (**A**) and infected monocyte-derived macrophages to *Rhizopus oryzae* after 6 h (**B**) and 16 h (**C**).

Macrophage aggregation was seen as early as a single minute and increased over time. After 6 h, 95% of the hyphae were surrounded by macrophage aggregates (Fig. 2).

#### Pattern of gene expression changes in the GPR78 gene

##### GPR78 gene expression in MDMs

**Fig 3:**
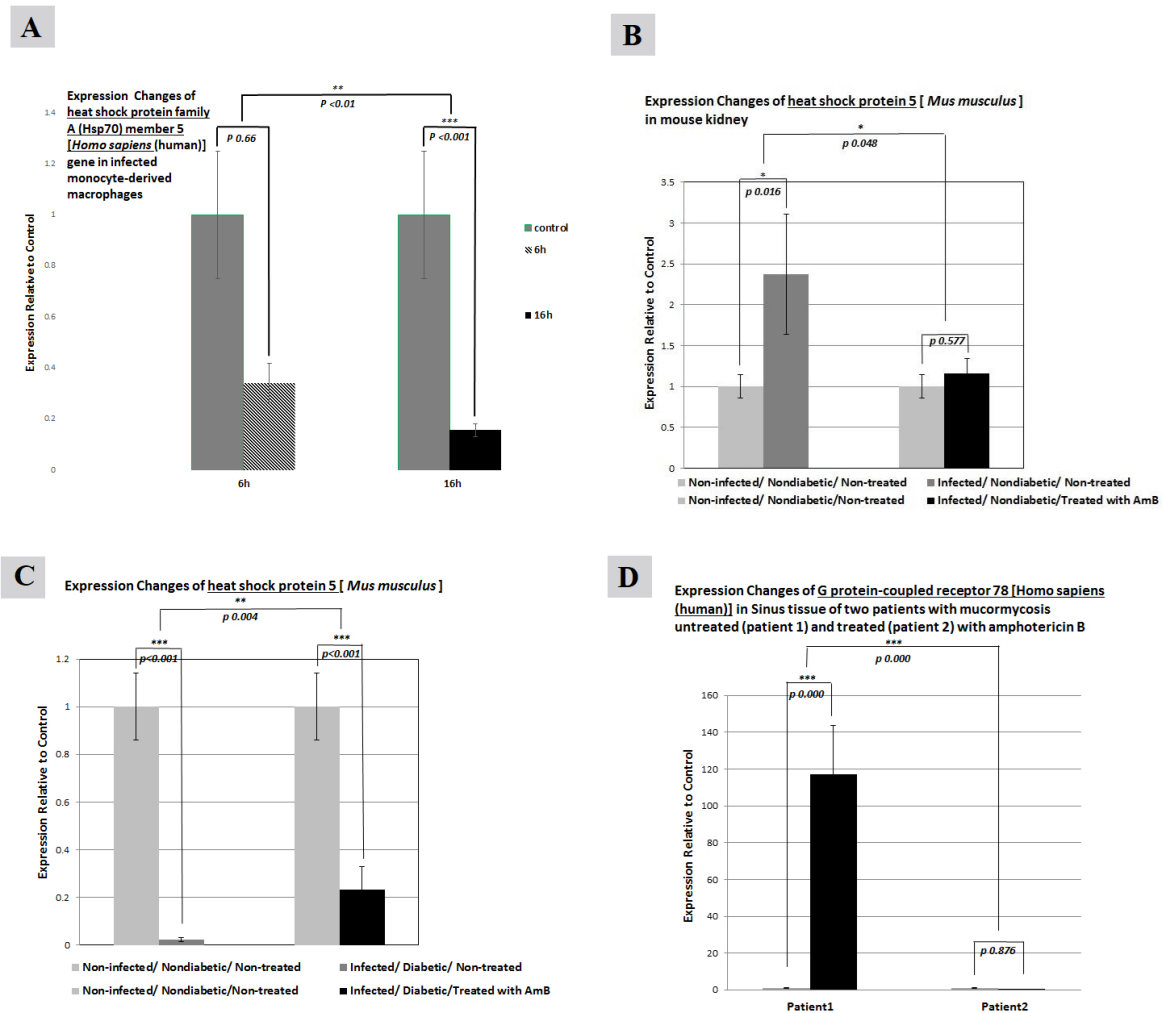
Relative expression changes of the *GPR78* gene in the MDMs infected with *R. oryzae* (**A**). All non-diabetic groups of mice (**B**). All diabetic and infected groups of mice (**C**). The sinus tissue of mucormycosis-infected patients (**D**). *P < 0.05, **P < 0.01, ***P < 0.001.

The fold changes in the GPR78 gene in the MDMs infected with *R. oryzae* after 6 h was about 1.346. However, the fold change of the same gene in MDMs infected with *R. oryzae* after 16 h was 2.522 (Fig. 3A).

##### GPR78 gene expression in all non-diabetic mice

As shown in Fig. 3B, for all infected non-diabetic mice that received LAmB treatment, the expression of the GPR78 gene was higher than that in the non-infected, non-diabetic mice without treatment.

##### GPR78 gene expression in all diabetic and infected mice

There is a clear trend of increasing expression of GPR78 in non-infected diabetic mice that did not receive amphotericin B (Fig. 3C). Also, there was upregulation of GPR78 gene expression in diabetic mice treated with LAmB and those without fungal invasion compared to the non-infected, non-diabetic mice and those without treatment.

##### GPR78 gene expression in the sinus tissue of mucormycosis-infected patients

The relative expression of the GPR78 gene was about 117 times higher in sinus tissue of mucormycosis-infected patients not treated with LAmB compared to control (the normal parts of the same infected tissue) (Fig. 3D). However, as it is clearly presented in the same diagram, GPR78 gene expression in patients with treatment has significantly decreased 87-fold compared to that of the control.

### Relative expression of miRNAs

#### Expression of hsa-miR-16-5p, hsa-miR-93-3p, and hsa-miR-335-5p genes in MDMs

**Fig 4:**
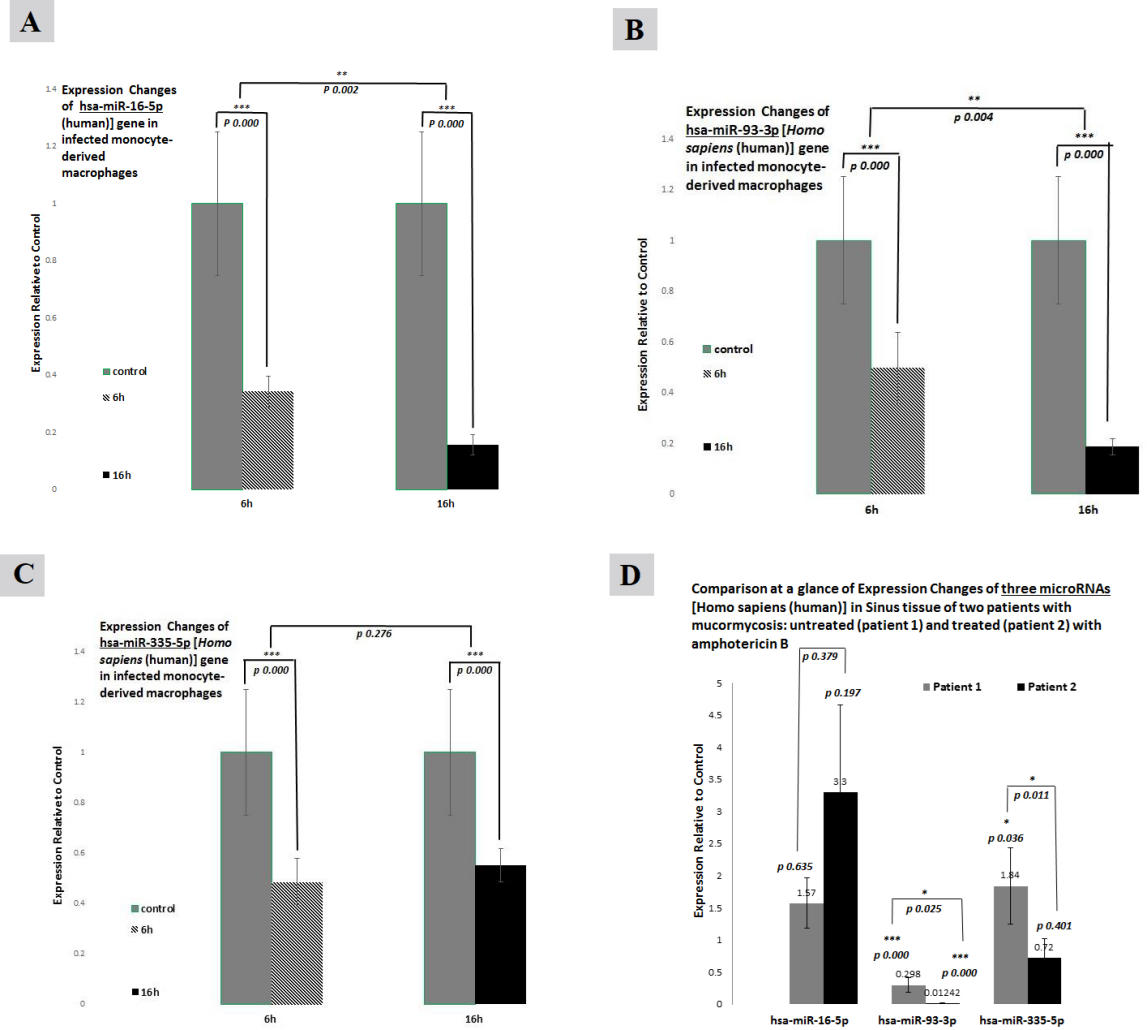
Relative expression changes of hsa-miR-16–5p (**A**), hsa-miR-93–3p (**B**), and hsa-miR-335–5p (**C**) genes in the MDMs infected with *R. oryzae*. The same three genes’ expression level in sinus tissue (**D**). *P < 0.05, **P < 0.01, ***P < 0.001.

The relative expression of *hsa-miR-16–5p*, *hsa-miR-93–3p*, and *hsa-miR-335–5p* genes was downregulated in the MDMs after 6 h of exposure with *R. oryzae*. Also, the same three genes’ expression level was decreased in the MDMs after 16 h of infection with *R. oryzae* (Fig. 4A through C).

#### Expression of hsa-miR-16-5p, hsa-miR-93-3p, and hsa-miR-335-5p in the sinus tissue of mucormycosis-infected patients

As shown in Fig. 4D, an increased expression of *hsa-miR-16–5p* and *hsa-miR-335–5p* genes was observed in the non-treated infected sinus tissue compared to control (the normal part of the same tissue). However, the expression of the *hsa-miR-93–3p* gene decreased. Additionally, for treated patients, *hsa-miR-16–5p* had a higher level of expression in comparison to control; however, the expression of the *hsa-miR-93–3p* and *hsa-miR-335–5p* genes was reduced.

#### Expression of mmu-miR-181b-5p gene in mice

**Fig 5:**
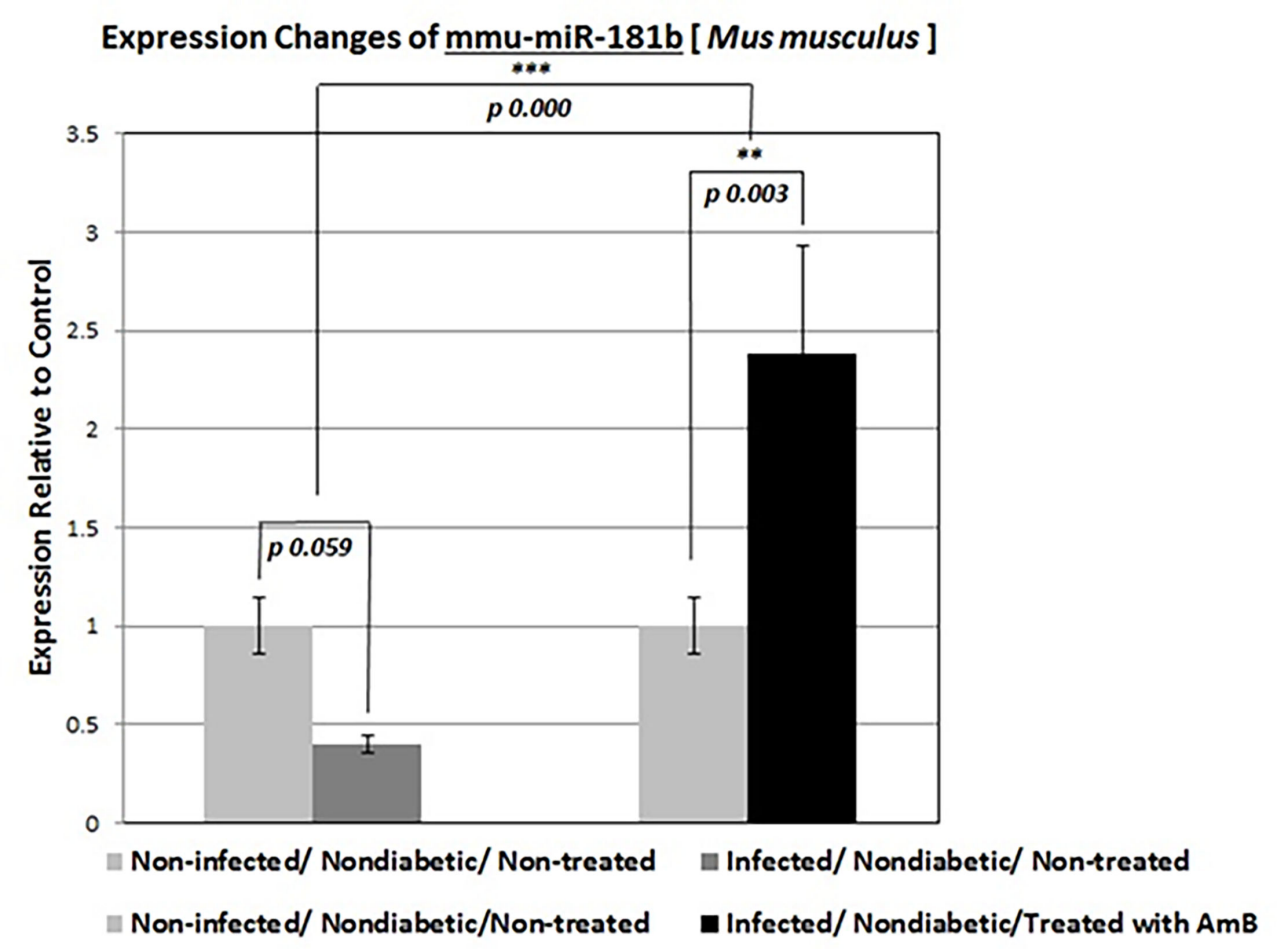
Relative expression changes of mmu-miR-181b-5p gene in all non-diabetic mice. **P < 0.01, ***P < 0.001.

**Fig 6:**
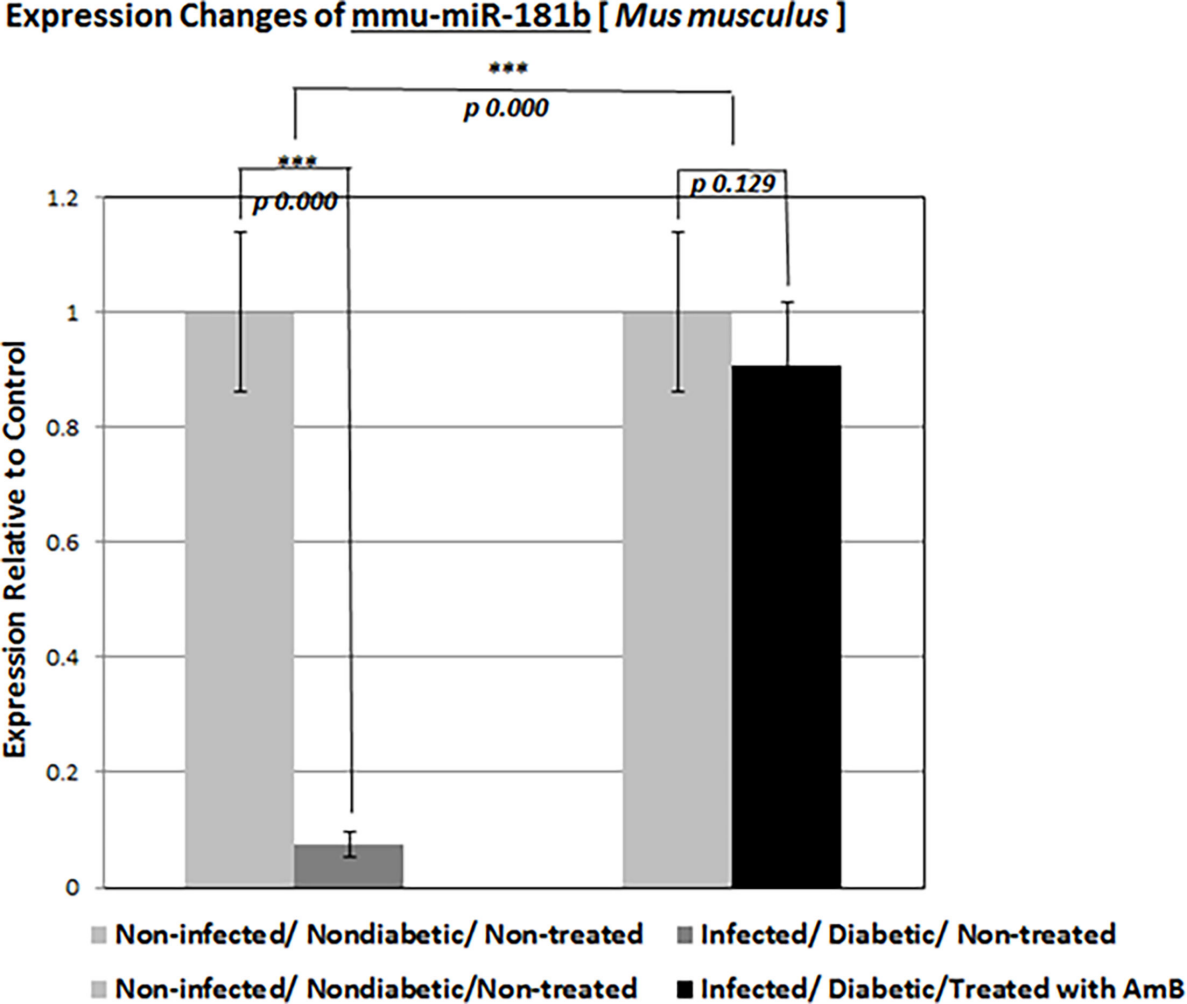
Relative expression changes of the mmu-miR-181b-5p gene in all diabetic mice infected with *Rhizopus oryzae*. ***P < 0.001

The relative expression of the mmu-miR-181-b gene was increased only in non-diabetic and *Rhizopus oryzae*-infected mice treated with amphotericin B, while it reduced in all groups. It was particularly, significantly reduced in the kidney tissue of all diabetic mice infected with *Rhizopus oryzae* (Fig. 5 and 6).

### Pattern of gene expression changes in CotH3, GPR87, hsa-miR-16-5p, hsa-miR-93-3p, and hsa-miR-335-5p

**Fig 7:**
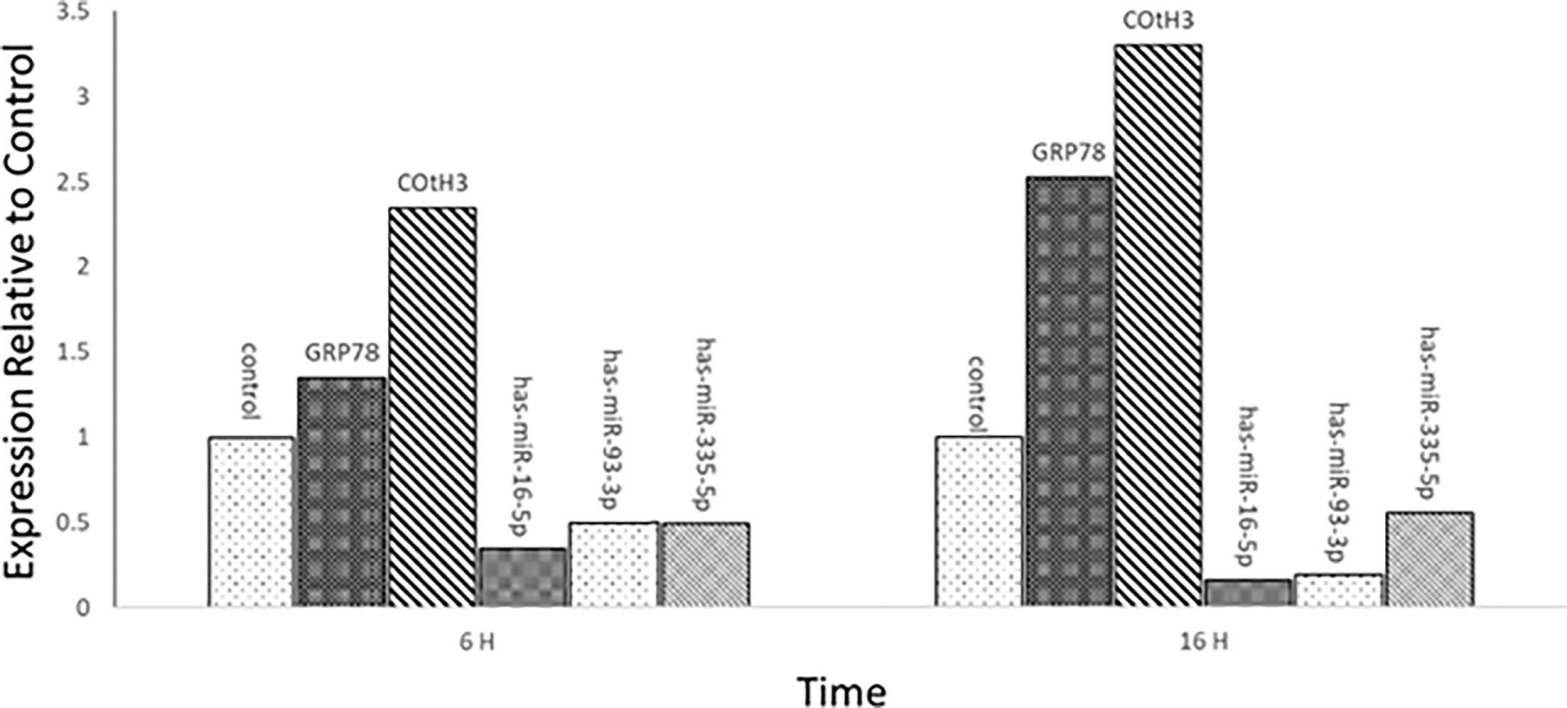
Gene expression changes pattern of CotH3, GPR87, hsa-miR-16–5p, hsa-miR-93–3p, and hsa-miR-335–5p in MDMs after 6 and 16 h of infection with *R. oryzae*.

The expression of CotH3 and GPR87 genes was upregulated after 6 and 16 h of infection with *R. oryzae*. However, the expression of the same genes increased significantly with time. Also, their target miRNAs displayed a significant reduction in expression over time since the infection (Fig. 7).

#### CotH3 gene expression in all non-diabetic and diabetic mice

Relative expression of the CotH3 gene was increased in the tissues of all diabetic and non-diabetic mice infected with *R. oryzae*, except for diabetic mice infected with *Rhizopus oryzae* treated with LAmB, which had a significant decrease in expression.

#### CotH3 gene expression in the sinus tissue of mucormycosis-infected patients

Changes in CotH3 gene expression were observed in sinus tissue that was prepared from untreated and LAmB-treated patients with mucormycosis in comparison with the normal margin of the same tissue (control). A 23-fold increase in gene expression occurred in the untreated mucormycosis-infected tissue of the patient compared to the normal margin tissue of the same patient (the control). On the other hand, in the treated patient, a significant 124-fold increase in gene expression occurred.

## DISCUSSION

Mucormycosis is the fourth most prevalent invasive fungal disease, following candidiasis, aspergillosis, and cryptococcosis (30). In the past, mucormycosis was perceived as a rare disease that is mainly linked with immunosuppressed patients including those living with HIV/AIDS, diabetes, and organ transplantation. However, with the recent advancement in diagnostic approaches, a wider spread of the disease has been confirmed (31). *In vitro* studies using cell lines as well as murine models have been successfully used to study pathogenesis and host-pathogen interactions and to evaluate therapy efficacy. Understanding host-pathogen interactions during the infection and the underlying mechanisms of host defense are critical for developing new prevention and treatment modalities, such as effective drugs and vaccines. This includes understanding the function of macrophages in inhibiting *R. oryzae* germination (32, 33). However, during the inhibition process, the fungal spores avert phagosome maturation by releasing melanin into the cell surface to maintain viability (34).

The findings of earlier studies on gene expression revealed that TLR2 receptor induction occurs immediately after *R. oryzae* and the primary inflammatory response of neutrophils (35). It has also been shown that infections of human nasal epithelial cells induced by *Mucor* spp. and *Rhizopus* spp. cause a robust inflammatory response, via the platelet-derived growth factor receptor B signaling pathway, leading to damage in host immune cells (11). Alternatively, *Rhizopus* spp. have been shown to induce an iron-restricting response in human macrophages, and its melanin prevents phagosome maturation and activates Akt/P13K signaling to suppress apoptosis pathways within the fungus. Therefore, the proliferation process remains active, and the fungus survives without the threat of cellular apoptosis (34). Previous studies demonstrated the expression of the CotH gene in *Rhizopus* spp. during interaction with host cells and proved its role in establishing the infection. Moreover, these genes can be targeted using PCR technology, which is more sensitive and more reliable for early detection of mucormycosis than conventional methods (36).

A study conducted by Gebremariam et al. has evaluated the effect of antibodies on CotH3, and the results revealed the protective role of polyclonal antibodies against mucormycosis in neutropenic and diabetic ketoacidosis mice (24). The study demonstrated that passive immunization by anti-CotH3 antibodies increased the migration of neutrophils into infected tissues through the Fc receptor and facilitated the killing of *Rhizopus delemar*. Furthermore, monoclonal antibodies produced against the CotH3 peptide protected immunosuppressed mice from mucormycosis caused by *R. delemar* and other Mucorales. These antibodies, coupled with antifungal drugs, showed a synergistic effect in preserving ketoacidotic mice. It has been demonstrated that GPR78—Mucorales species receptor present on the surface of host endothelial cells—plays an essential role in invasion and damage associated with this fungus (24).

### GRP78 and CotH3 gene expression in infected macrophages

In this study, a significant increase was observed in the CotH3 gene expression in most *ex vivo*, *in vivo*, and clinical samples. This gene is one of the essential fungal factors for host cell invasion. Therefore, it is noticeable that gene expression and host cell damage were higher with prolonged exposure to the fungus. These results are consistent with those obtained by Gebremariam et al., who reported an increased expression of the CotH3 gene in Chinese hamster ovary cells infected with *R. oryzae* hypha (10). The higher expression of this gene proves its importance in Mucorales pathogenesis. As for the GPR78 gene, results indicated an increased expression in the tested samples, and this increase is positively associated with the period of time since infection. Since the CotH protein is a ligand to the GPR78 receptor, as suggested by Ibrahim et al. (37), the increase in the expression of the two genes could have been caused by macrophage cell invasion with fungal hypha (37).

### GRP78 and CotH3 expression in renal tissues of non-diabetic and diabetic mice infected with mucormycosis and not treated with LAmB

In the current study, a 20 times increase was observed in the expression of CotH3 in diabetic mice compared to non-diabetic mice. This indicates that diabetes facilitates fungal hypha invasion and mycelial expansion, which is possibly reflected in elevated gene expression. Interestingly, the group of diabetic mice infected with mucormycosis and not treated with LAmB showed the highest level of CotH3 expression among all groups of mice being studied, indicating a correlation between diabetes and CotH3 expression. This is very alarming for diabetic people as this result suggests that they are at higher risk of infection, which is further supported by the particularly high prevalence of mucormycosis among diabetic patients (38). Therefore, effective prevention and case management measures are urgently needed, particularly for people living with diabetes. Similarly, the GPR78 expression level in infected non-diabetic mice that did not receive LAmB treatment was higher than that in similar mice treated with LAmB. This difference could be attributed to the fact that LAmB treatment lessened the stress level in endothelial renal tissues of mice that received treatment.

### GRP78 and CotH3 expression in renal tissues of diabetic infected mice with and without LAmpB treatment

According to the results of our study, diabetic and infected mice that underwent treatment exhibited a significant reduction in CotH3 gene expression when compared to the mice that did not undergo treatment. In addition, the GPR78 gene expression level in non-infected diabetic mice without liposomal amphotericin B treatment was higher compared to treated ones. Interestingly, only the group of infected diabetic mice with treatment showed a decrease in GPR78 gene expression among all the examined groups. It suggested that elevated stress due to fungal infection has been subdued by the administration of liposomal amphotericin B, which is in turn causing a reduction in gene expression.

### Clinical samples of mucormycosis

In case of clinical mucormycosis, the higher level of blood sugar in non-treated patients could explain the effect of this factor on fungal growth and the subsequent increase in CotH3 expression. Moreover, considering the high expression of this gene in treated patients, it can be concluded that the presence of necrosis (the primary characteristic of Mucorales) in infected areas might have reduced the impact of antifungal treatment on the infected tissues. Considering that sufficient drug delivery to tissues needs intact blood vessels, the marked tissue destruction in necrosed areas might facilitate fungal growth due to the truncated amphotericin B delivery therein. Furthermore, the expression of the GPR78 gene in the sinus tissue of diabetic patients infected with untreated mucormycosis was higher than that in the normal tissue of the same patients. However, samples from similar patients with LAmB treatment showed a decrease in expression. Although an increase in gene expression was expected in diabetic patients, surprisingly, the expression level was reduced due to necrosis in infected tissues and dead cells (39).

### Analysis of miRNAs targeting GPR78 in infected macrophages

The expression of hsa-miR-16–5p, hsa-miR-93–3p, and hsa-miR-335–5p genes among the infected samples has decreased during 16 h of infection compared to control. Whereas one of the probable targets of hsa-miR-16–5p is the ATK gene associated with the GPR78 signaling pathway, the increased expression of GPR78 after 6 and 16 h of infection decreased the hsa-miR-16–5p expression through a suppressive effect. Alternatively, the increased CotH3 gene expression after 6 and 16 h of the infection indicates the preservation of the fungus’s ability to grow into hyphae. Moreover, it is expected that during the phagocytosis procedure, the expression of hsa-miR-16–5p, hsa-miR-93–3p, and hsa-miR-335–5p interacts with the regulatory intracellular signaling pathways, such as MyD88 and TLR signaling, which control gene expression through transcription factors (20). Moreover, the excessive hypha growth and development in prolonged exposure to macrophages might have caused a reduction in miRNA expression, possibly to maintain the survival of the fungus.

Our study provided a combination of gene expression and miRNA analyses in both fungal and host cells during *in vitro* and *in vivo* infection and presented a framework for understanding the molecular pathogenesis of mucormycosis. The use of fresh biological samples from both mucormycosis patients and animal models can be considered an advantage of this study. In addition, the current study was designed to further understand the interaction between CotH and GPR78, which might pave the way to achieve new therapeutic methods against fungal infection and propose their application as biomarkers at specific stages of infection development by combining bioinformatics, biochemical, and genetic approaches. Moreover, this study investigated the expression of miRNA and highlighted its role as a therapeutic approach against the fungi. Furthermore, for a better understanding of the interactions between miRNAs, CotH, and GPR78, studies tailored specifically to determine and establish novel treatment approaches are highly recommended. One of the limitations of the present study was the small clinical sample size. So, large-scale studies are needed to confirm our findings.

Although we tried our best, this study has some limitations that warrant consideration. The small sample size of clinical cases may restrict the generalizability of the findings, emphasizing the need for larger-scale studies. Additionally, while murine models and *ex vivo* analyses provided valuable insights, they may not fully capture the complexity of human infections. Further investigations involving diverse patient populations and advanced *in vivo* models are essential to bridge these gaps.

Future research should focus on developing targeted therapeutic strategies to inhibit the interaction between CotH3 and GPR78, such as monoclonal antibodies or small-molecule inhibitors. Moreover, miRNAs like hsa-miR-16–5p, hsa-miR-93–3p, and hsa-miR-335–5p hold promise as early diagnostic biomarkers and potential therapeutic tools for mucormycosis. Enhancing drug delivery systems, particularly for necrotic tissues, should also be prioritized to improve treatment efficacy.

### Conclusion

The diabetic condition influences interaction between the ligand and receptor within a particular host. Diabetes caused an elevation in the level of expression of the GPR78 gene in line with an increase in the level of glucose, iron, and ketone bodies, leading to a higher fungal growth rate. In addition, acidotic conditions in diabetic individuals indirectly influence phagocytosis and pathogen growth through iron release from transferrin and increased CotH3 and GPR78 expression. This highlights the critical role of diabetes as an underlying condition with regard to a higher risk of mucormycosis infection. Furthermore, this indicates the need for further studies on this subject to discover effective management modalities. In this study, the increased gene expression levels of CotH3 and GPR78 in infected macrophages highlight the subcellular pathogen-macrophage interactions, and the prolonged encounter leads to changes in the expression level of these genes and a reduction in miRNAs (hsa-miR-16–5p, hsa-miR-335–5p, and hsa-miR-93–3p) targeting GPR78. These microRNAs can be used as invaluable biomarkers that are particularly useful for the early detection and following up on the treatment processes.

## Data Availability

Data sharing is not applicable. Data are available on request due to restrictions (e.g., privacy or ethical). The data presented in this study are available on request from the corresponding author.
